# Social and professional stigma during COVID-19 among healthcare workers in a Tunisian hospital

**DOI:** 10.1192/j.eurpsy.2023.1250

**Published:** 2023-07-19

**Authors:** N. Mechergui, N. Chaouech, I. Youssef, M. Mersni, D. Brahim, G. Bahri, S. Ernez, H. Ben Said, N. Ladhari

**Affiliations:** 1 Charles Nicolle hospital; 2 Habib Thameur hospital, Tunis, Tunisia

## Abstract

**Introduction:**

Healthcare workers (HCWs) are on the front line in the management of the COVID-19 pandemic. They are at higher risk of acquiring SARS-CoV2 infection and might transmit the virus to other person or their family members. All these gave rise to stigma toward society, family, and HCWs.

**Objectives:**

to measure social and professional stigma and guilty feelings among HCWs in a Tunisian hospital.

**Methods:**

A cross-sectional study using a questionnaire (sociodemographic and professional characteristics and three questions (yes/no) related to social and professional stigma and guilty feelings) was conducted from September 1 to December 31, 2020, at Charles Nicolle hospital of Tunis. The survey was distributed among HCWs consulting the department of occupational health after a COVID-19 infection.

**Results:**

A total of 259 HCWs were included in the study. The mean age was 41±10 years with a sex ratio of 0.25. The HCWs were married in 66.8% of cases. The professional categories were represented mainly by nurses in 33.5% of cases followed by technicians and physicians in 26.2% and 17.4% of cases respectively. The average professional seniority was 13 years (min=1 year; max=13 years).

The social stigma was reported by 30.5% and professional stigma by 20.1%. The stigmatized population was predominantly female (86%), and the proportion of married people was 63%. The predominant professional category was nurses (36%) followed by senior technicians and workers (25% and 16% respectively). The average length of employment was 13 years. Guilt was felt by 57.1% of the cases.

**Conclusions:**

Based on the results of this study, the social and professional stigma during COVID-19 among HCWs was significant as well as the feeling of guilt. This may have an adverse impact on HCWs’ mental health.

**Disclosure of Interest:**

None Declared

